# Editorial

**Published:** 1916-07

**Authors:** 


### EDITORIAL

The Atlanta Journal-Record of Medicine has its Business Office at 23 West Alabama Street where all business communications should be sent.

Remittances are payable to The Journal-Record of Medicine.

Concerning Original Communications and Editorial matters, address Richard R. Daly, M. D., 708 Empire Life Building, Atlanta, Ga.

Reprints of Original Articles will be furnished at cost price. Requests for them had best be made on the Manuscript.

Upon request, we will present, postpaid, to each contributor of an original article, twenty copies of The Journal-Record of Medicine containing such article.

### THE EPIDEMIC.

New York City is fighting with all its forces to control the spread of Infantile Paralysis that has been threatening the city and the nation for several weeks. The authorities have been calling upon the resources of the entire Profession and the invitation sent to specially skilled men all over the Country to assemble at New York and assist in gaining control of the disease shows that they intend nothing shall be left undone.

Thus far the ravages have been confined to the metropolitan district, but the history of the disease tells us that it has occurred in several places throughout the land and that many years ago, it had its sway in Louisiana. Whether there is something in the conditions around New York that cause the disease at this time, we can not say. No one knows how the disease is propagated and carried, but we do know that it may arise in other places.

The people are frightened and the inquiries that come constantly to us as to whether children may safely be moved from one locality to another show a reasonable parental anxiety. That we can not answer them definitely is to be regretted, but at the same time we can be reassuring. Advice as to cleanliness and sanitation will be accepted and when taken it will help any child to increase his resistance to all disease and it will tend to protect him from needless exposure. Many of the traditionally held ideas as to prophylaxis can well be dispelled. The shutting of the child away from night air so that disease shall not reach him through the windows is one of these. It is time also to give the parents instructions as to what good food and careful bathing mean and to set aside many home remedies and old women's treatments that are never applied except to the child's detriment.

It is not too much to insist upon clean food, clean bodies and clean playgrounds for the children. They should have these always, but we have to admit that they do not. If they are given now, there will be far less danger from poliomyelitis than under the back yard methods.

The somewhat amusing discoveries in Oyster Bay of the dirtiest kinds of back yards and the most primitive sanitary surroundings in the home city of omniscience—at the well spring of beneficence in popular guidance—will show us that we are not alone in having something to do that should have been done a long time ago. The citizens there have started house cleaning in earnest and we must admit that they are doing it because they discovered what they thought menaced their children. It was not entirely because they were found out and laughed at. Let every one of us get after his own back yard—after his own city and clean them for the sake of the children. There can be no mistake in getting clean and when the investigators have found the reason for the disease, we shall have empirically traversed half the distance toward completing their work.

The men who have been invited to serve upon New York's Committee are known everywhere and we can have confidence that if they accept and serve, the service will be reliable and great indeed.

---

### TERMINOLOGY.

In the July "Journal of Nervous and Mental Diseases" there is an article by Augusta F. Bronner, Ph.D., entitled "What

Do Psychiatrists Mean?" It is illuminating and suggestive in many ways.

Regarding first the peculiar and special conditions of the subject matter of the article, the writer shows that we are all careless in the use of words and that we say one thing when, very often we mean another. The confusion that results from this is injurious to the patients and reflects anything but credit upon ourselves. It is all very well in uncomplicated cases to have a layman say: "Well, I fancy the Doctor meant so and so," and thus put his own interpretation upon our decisions, but where life and liberty may be at stake, it behooves us to say things so clearly that none but the right interpretation can be made. When we write prescriptions, we want the druggist to put in exactly what we order, or, failing in ability to do that, he is to call us up before using his judgment. Yet we allow the unskilled layman to carry the impression that we have said things as he wants them and to lay the responsibility upon us when things go wrong.

The verbosity of legal documents has long been a matter of ridiculous annoyance to most of us; it should be cleared and clarified, but at the same time medico-legal matters have much to remove from their pathway before the doctors can well get at the rubbish of the lawyers.

The terms "idiot" and "imbecile" are often confused by the general practitioner and they have been misused by the specialists who have not taken time for thorough analysis of cases.

"Illusion, delusion" and "hallucination" are often mixed and confused by the careless speaker and the result is that when explanations are forced in cross examinations the testimony which really has inherent value may be disqualified by an appearance of inexact thought and of little study. Nothing can make a man look inexpert more than the showing that he does not know how to use his tools. Medical nomenclature is not difficult to acquire, but it does take time and when once secured it is an evidence of a man's method of thought. It is the tool which shows in the using whether it is handled by a master or a bungler; whether it is an instrument that is carefully and accurately guided, or whether it is a force that sways and controls a careless man against his will.

The use of such words as "bilious" and "catarrh" by our patients leads us into carelessness because we can not stop to explain the true situation every time someone asks about it. Just what "bilious" means no one knows—not even the user when he asks if his friend is not suffering with biliousness. Catarrh has a definite meaning but no layman ever understood it without taking careful thought. The taking of careful thought is not the layman's method when it comes to medical and hygienic methods. He simply wants some words. It is all very well to let him have them except that he forms opinions upon such insecure and incorrect bases and then he gets in the way of sanitation because he thinks he knows more about it than he really does.

Words are gems that may be gloriously beautiful when correctly assembled; the clusters convey impressions that always persist and serve as guides through life's perplexities. The man who realizes their power and his own responsibility will be careful in their use.

